# (*E*)-1-Ferrocenyl-3-(3-nitro­phen­yl)prop-2-en-1-one

**DOI:** 10.1107/S1600536808027815

**Published:** 2008-09-06

**Authors:** Yong-Hong Liu, Jun Ye, Xiao-Lan Liu, Wen-Long Liu, Yao-Cheng Shi

**Affiliations:** a College of Chemistry and Chemical Engineering, Yangzhou University, Yangzhou 225002, People’s Republic of China

## Abstract

In the title compound, [Fe(C_5_H_5_)(C_14_H_10_NO_3_)], one cyclo­penta­diene ring is substituted and one is unsubstituted. The two rings are almost parallel and are eclipsed and ordered. The conjugated substituent is slightly twisted with respect to the cyclo­penta­diene ring. The crystal structure contains four inter­molecular C—H⋯O hydrogen-bonds in the range 3.324 (3)–3.539 (3) Å and one *π*(aryl ring)–*π* (Cp ring) stacking inter­action with a ring–centroid distance of 3.894 (2) Å.

## Related literature

For related literature, see: Allen *et al.* (1987 ▶); Bernstein *et al.* (1995 ▶); Harrison *et al.* (2006 ▶); Kealy & Pauson (1951 ▶); Liang *et al.* (1998 ▶); Liu *et al.* (2001 ▶, 2003 ▶, 2008 ▶); Mrisra & Tenari (1973 ▶); Shi *et al.* (2004 ▶). Yarishkin *et al.* (2008 ▶); Zhai *et al.* (1999 ▶).
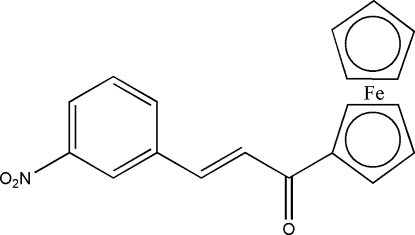


## Experimental

### 

#### Crystal data

[Fe(C_5_H_5_)(C_14_H_10_NO_3_)]
*M* *_r_* = 361.17
Triclinic, 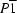

*a* = 5.8691 (7) Å
*b* = 10.8636 (12) Å
*c* = 12.6193 (14) Å
α = 77.038 (2)°
β = 81.562 (2)°
γ = 83.565 (2)°
*V* = 772.99 (15) Å^3^
*Z* = 2
Mo *K*α radiation
μ = 0.99 mm^−1^
*T* = 296 (2) K
0.30 × 0.30 × 0.20 mm

#### Data collection

Bruker SMART 1000 CCD diffractometer
Absorption correction: multi-scan (*SADABS*; Bruker, 2007 ▶) *T*_min_ = 0.755, *T*_max_ = 0.826
5617 measured reflections
2686 independent reflections
2462 reflections with *I* > 2σ(*I*)
*R*_int_ = 0.058

#### Refinement

*R*[*F* ^2^ > 2σ(*F* ^2^)] = 0.043
*wR*(*F* ^2^) = 0.116
*S* = 1.10
2686 reflections
217 parameters
H-atom parameters constrained
Δρ_max_ = 0.73 e Å^−3^
Δρ_min_ = −0.52 e Å^−3^

### 

Data collection: *SMART* (Bruker, 2007 ▶); cell refinement: *SAINT* (Bruker, 2007 ▶); data reduction: *SAINT*; program(s) used to solve structure: *SHELXS97* (Sheldrick, 2008 ▶); program(s) used to refine structure: *SHELXL97* (Sheldrick, 2008 ▶); molecular graphics: *PLATON* (Spek, 2003 ▶); software used to prepare material for publication: *SHELXTL* (Sheldrick, 2008 ▶).

## Supplementary Material

Crystal structure: contains datablocks I, global. DOI: 10.1107/S1600536808027815/om2257sup1.cif

Structure factors: contains datablocks I. DOI: 10.1107/S1600536808027815/om2257Isup2.hkl

Additional supplementary materials:  crystallographic information; 3D view; checkCIF report

## Figures and Tables

**Table 1:** Hydrogen-bond geometry (Å, °)

*D*—H⋯*A*	*D*—H	H⋯*A*	*D*⋯*A*	*D*—H⋯*A*
C7—H7⋯O3^i^	0.93	2.54	3.324 (3)	143
C14—H14⋯O2^i^	0.93	2.67	3.377 (3)	134
C3—H3⋯O1^ii^	0.93	2.66	3.278 (3)	124
C17—H17⋯O1^iii^	0.93	2.68	3.539 (3)	154

Symmetry codes: (i) 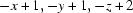; (ii) 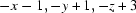; (iii) 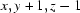.

**Fig. 1.**
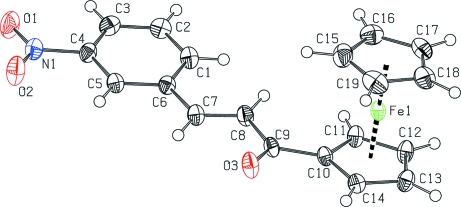
The molecular structure of the title compound, showing 50% probability displacement ellipsoids.

**Fig. 2.**
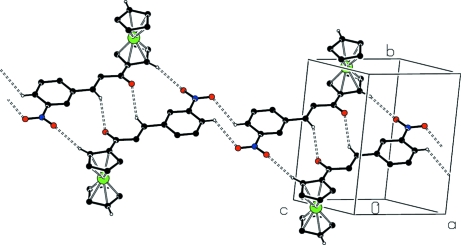
Part of the crystal structure of the title compound, showing the formation of a R22(10)R22(12)R22(12)R22(12) hydrogen bonded chain along a axis, which is built by three C—H···O inter-molecular hydrogen bonds (dashed lines). For the sake of clarity, H atoms not involved in hydrogen bonding have been omitted.

**Fig. 3.**
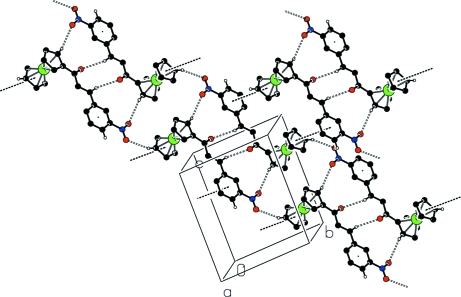
Part of the crystal structure of the title compound, showing the formation of a hydrogen bonded R44(16) chain via C—H···O and pi (aryl ring)···pi (Cps ring) inter-molecular hydrogen bonds (dashed lines) along c axis,. For the sake of clarity, H atoms not involved in hydrogen bonding have been omitted.

## supplementary crystallographic information

### Comment

α, β-unsaturated ketones are important as intermediates in many addition reactions and they are also used widely in synthesizing of spice and medicament and materials (Mrisra *et al.*, 1973; Zhai *et al.*,1999; Liu *et al.*, 2001, 2003; Yarishkin *et al.*, 2008). Since the discovery of ferrocene (Kealy & Pauson, 1951), ferrocene has played an important role in the development of electronic structures of organometallic compounds and materials chemistry. A considerable number of ferrocene derivatives have been prepared directly or indirectly from ferrocene and their properties have been extensively studied. As part of our search for new biological active compounds (Liang *et al.*, 1998; Shi *et al.*, 2004; Liu *et al.*, 2008), we report herein the synthesis and crystal structure of the tltle compound.

The molecule of the title compound exists as the most stable configuration of (*E)*-isomer (Scheme 1, Fig.1, Table 1). The Cps ring is connected to the phenyl group through the C6—C7=C8—C9—C10 chain with the C=C bond length being 1.330 (3) Å and the three single C_sp2_—C_sp2_ bond lengths ranging from 1.472 (3) to 1.477 (3) Å, which are the same with the result of our early works (Liu *et al.*, 2008). This rang compares well with the statistical values for such bond lengths in conjugated C=C—C(=O)—C system [1.464 (18) Å] and for C_sp2_—C_aryl_ bonds (lower quartile 1.472 Å) (Allen *et al.*, 1987). The C9=O3 and C4—N1 bond distances are 1.226 (3) and 1.473 (3) Å, The Cp and Cps rings are nearly parallel [dihedral angle 0.99 (11)°]. The dihedral angle between the benzene ring and Cps ring is 6.6 (10)°, which is in agreement with the literature (Harrison *et al.*, 2006). The nitro group is well ordered and makes a dihedral angle of 4.57 (3)° with respect to the benzene ring.

In its packing structure, along *b* axis two neighboring molecules are linked into *R_2_^2^(12)R_2_^2^(12)R_2_^2^(12)* (Bernstein *et al.*, 1995) dimer by two pairs of C14–H14(Cps)···O2(nitro) and C7–H7···O3=C inter-molecular hydrogen-bonds and the two neighboring dimers are linked into *R_2_^2^(10)* ladder-shape by two C3–H3(aryl)···O1(nitro) inter-molecular hydrogen-bonds, thus forming cross edge-fused *R_2_^2^(10)R_2_^2^(12)R_2_^2^(12)R_2_^2^(12)* sheet (Fig. 2, Table 2). At same time, along *c* axis the two neighboring dimers linked into *R_4_^4^(16)* chains and the neighboring chains above and below are assemble into a block *via π*(aryl ring)···*π*(Cp ring) inter-molecular stacking interactions (the corresponding ring-centroid separation is 3.894 (2) Å) (Fig. 3). All of the above mentioned inter-molecular hydrogen-bonds link the molecules into a three-dimensional structure of considerable complexity.

### Experimental

Acetylferrocene (1.98 g, 0.01 mol) in ethanol (25 ml) was mixed with 3-nitrobenzaldehyde (1.51 g, 0.01 mol) in ethanol (25 ml) and the mixture was treated with an aqueous solution (20 ml) of potassium hydroxide (20 ml, 5%). The resulting mixture was stirred well and left for 24 h, and the solid product was collected by filtration and dried. Crystals of the product were obtained from ethanol recrystallization (yield 80%; m.p. 463 K). Analysis, found (calculated) for C_19_H_15_O_3_NFe (%): C 63.16 (63.29), H 4.16 (4.12), N 3.88 (3.65).

### Refinement

After their location in a difference map, all H atom were fixed geomerically at ideal positions and allowed to ride on the parent C atom.with C—H distances of 0.93 Å(CH) or 0.98 Å (ferrocenyl), and with *U*_iso_(H) values of 1.2Ueq(C).

### Crystal data

**Table:**

[Fe(C_5_H_5_)(C_14_H_10_NO_3_)]	*Z* = 2
*M**_r_* = 361.17	*F*(000) = 372
Triclinic, *P*1	*D*_x_ = 1.552 Mg m^−^^3^
Hall symbol: -p 1	Melting point: 463 K
*a* = 5.8691 (7) Å	Mo *K*α radiation, λ = 0.71073 Å
*b* = 10.8636 (12) Å	Cell parameters from 4689 reflections
*c* = 12.6193 (14) Å	θ = 2.3–27.5°
α = 77.038 (2)°	µ = 0.99 mm^−^^1^
β = 81.562 (2)°	*T* = 296 K
γ = 83.565 (2)°	Block, red
*V* = 772.99 (15) Å^3^	0.30 × 0.30 × 0.20 mm

### Data collection

**Table:**

Bruker SMART 1000 CCD diffractometer	2686 independent reflections
Radiation source: fine-focus sealed tube	2462 reflections with *I* > 2σ(*I*)
graphite	*R*_int_ = 0.058
ω scans	θ_max_ = 25.0°, θ_min_ = 1.7°
Absorption correction: multi-scan (SADABS; Bruker,2007)	*h* = −6→6
*T*_min_ = 0.755, *T*_max_ = 0.826	*k* = −12→12
5617 measured reflections	*l* = −15→15

### Refinement

**Table:**

Refinement on *F*^2^	Primary atom site location: structure-invariant direct methods
Least-squares matrix: full	Secondary atom site location: difference Fourier map
*R*[*F*^2^ > 2σ(*F*^2^)] = 0.043	Hydrogen site location: inferred from neighbouring sites
*wR*(*F*^2^) = 0.116	H-atom parameters constrained
*S* = 1.10	*w* = 1/[σ^2^(*F*_o_^2^) + (0.0764*P*)^2^ + 0.0618*P*] where *P* = (*F*_o_^2^ + 2*F*_c_^2^)/3
2686 reflections	(Δ/σ)_max_ = 0.001
217 parameters	Δρ_max_ = 0.73 e Å^−^^3^
0 restraints	Δρ_min_ = −0.52 e Å^−^^3^

### Special details

**Table:**

Geometry. All e.s.d.'s (except the e.s.d. in the dihedral angle between two l.s. planes) are estimated using the full covariance matrix. The cell e.s.d.'s are taken into account individually in the estimation of e.s.d.'s in distances, angles and torsion angles; correlations between e.s.d.'s in cell parameters are only used when they are defined by crystal symmetry. An approximate (isotropic) treatment of cell e.s.d.'s is used for estimating e.s.d.'s involving l.s. planes.
Refinement. Refinement of *F*^2^ against ALL reflections. The weighted *R*-factor *wR* and goodness of fit *S* are based on *F*^2^, conventional *R*-factors *R* are based on *F*, with *F* set to zero for negative *F*^2^. The threshold expression of *F*^2^ > σ(*F*^2^) is used only for calculating *R*-factors(gt) *etc*. and is not relevant to the choice of reflections for refinement. *R*-factors based on *F*^2^ are statistically about twice as large as those based on *F*, and *R*- factors based on ALL data will be even larger.

### Fractional atomic coordinates and isotropic or equivalent isotropic displacement parameters (Å^2^)

**Table:**

	*x*	*y*	*z*	*U*_iso_*/*U*_eq_	
Fe1	0.23081 (5)	0.97010 (3)	0.73142 (2)	0.02174 (17)	
O1	−0.2073 (4)	0.3484 (2)	1.52513 (15)	0.0494 (6)	
O2	0.1462 (3)	0.3394 (2)	1.44848 (15)	0.0441 (5)	
O3	0.5123 (3)	0.67292 (17)	0.89307 (14)	0.0349 (4)	
N1	−0.0581 (4)	0.37596 (19)	1.44662 (16)	0.0315 (5)	
C1	−0.2608 (4)	0.6133 (2)	1.1575 (2)	0.0279 (5)	
H1A	−0.3072	0.6658	1.0941	0.033*	
C2	−0.4195 (5)	0.5856 (2)	1.2499 (2)	0.0307 (6)	
H2	−0.5711	0.6210	1.2482	0.037*	
C3	−0.3572 (4)	0.5063 (2)	1.3449 (2)	0.0295 (6)	
H3	−0.4650	0.4867	1.4067	0.035*	
C4	−0.1309 (5)	0.4573 (2)	1.34520 (18)	0.0265 (5)	
C5	0.0351 (4)	0.4822 (2)	1.25377 (18)	0.0238 (5)	
H5	0.1860	0.4459	1.2561	0.029*	
C6	−0.0307 (4)	0.5631 (2)	1.15843 (18)	0.0237 (5)	
C7	0.1479 (4)	0.5950 (2)	1.06407 (18)	0.0254 (5)	
H7	0.2918	0.5499	1.0683	0.030*	
C8	0.1221 (4)	0.6825 (2)	0.97330 (19)	0.0286 (5)	
H8	−0.0198	0.7294	0.9673	0.034*	
C9	0.3099 (4)	0.7080 (2)	0.88167 (19)	0.0248 (5)	
C10	0.2434 (4)	0.7779 (2)	0.77460 (18)	0.0227 (5)	
C11	0.0152 (4)	0.8290 (2)	0.74917 (19)	0.0246 (5)	
H11	−0.1220	0.8188	0.7963	0.030*	
C12	0.0394 (4)	0.8981 (2)	0.63821 (19)	0.0271 (5)	
H12	−0.0803	0.9411	0.6003	0.033*	
C13	0.2780 (4)	0.8900 (2)	0.59541 (19)	0.0279 (5)	
H13	0.3406	0.9267	0.5248	0.033*	
C14	0.4033 (4)	0.8164 (2)	0.67886 (18)	0.0232 (5)	
H14	0.5623	0.7965	0.6725	0.028*	
C15	0.3201 (6)	1.0342 (3)	0.8590 (2)	0.0383 (6)	
H15	0.3604	0.9838	0.9245	0.046*	
C16	0.0932 (5)	1.0845 (2)	0.8380 (2)	0.0344 (6)	
H16	−0.0410	1.0732	0.8872	0.041*	
C17	0.1083 (5)	1.1552 (2)	0.7285 (2)	0.0351 (6)	
H17	−0.0151	1.1979	0.6932	0.042*	
C18	0.3411 (5)	1.1497 (2)	0.6821 (2)	0.0369 (6)	
H18	0.3982	1.1887	0.6112	0.044*	
C19	0.4750 (5)	1.0740 (3)	0.7625 (3)	0.0379 (6)	
H19	0.6340	1.0544	0.7535	0.046*	

### Atomic displacement parameters (Å^2^)

**Table:**

	*U*^11^	*U*^22^	*U*^33^	*U*^12^	*U*^13^	*U*^23^
Fe1	0.0215 (2)	0.0200 (2)	0.0219 (2)	−0.00606 (15)	−0.00413 (15)	0.00257 (15)
O1	0.0496 (13)	0.0551 (13)	0.0287 (10)	0.0023 (10)	0.0098 (9)	0.0095 (9)
O2	0.0332 (11)	0.0566 (12)	0.0347 (10)	−0.0037 (9)	−0.0080 (8)	0.0091 (9)
O3	0.0232 (10)	0.0366 (10)	0.0364 (10)	−0.0013 (7)	−0.0054 (7)	0.0103 (8)
N1	0.0347 (13)	0.0308 (11)	0.0270 (11)	−0.0051 (9)	−0.0042 (9)	−0.0007 (9)
C1	0.0252 (13)	0.0266 (12)	0.0314 (12)	−0.0070 (10)	−0.0082 (10)	0.0001 (10)
C2	0.0280 (14)	0.0303 (13)	0.0347 (13)	−0.0055 (10)	−0.0041 (10)	−0.0074 (10)
C3	0.0285 (14)	0.0316 (13)	0.0292 (13)	−0.0116 (10)	0.0027 (10)	−0.0081 (10)
C4	0.0356 (14)	0.0225 (12)	0.0221 (12)	−0.0110 (10)	−0.0032 (10)	−0.0020 (9)
C5	0.0210 (12)	0.0210 (11)	0.0293 (12)	−0.0056 (9)	−0.0045 (9)	−0.0022 (9)
C6	0.0274 (13)	0.0198 (11)	0.0243 (12)	−0.0091 (9)	−0.0034 (9)	−0.0022 (9)
C7	0.0223 (12)	0.0244 (12)	0.0288 (12)	−0.0054 (9)	−0.0061 (9)	−0.0010 (9)
C8	0.0288 (13)	0.0246 (12)	0.0280 (12)	0.0003 (10)	−0.0028 (10)	0.0020 (9)
C9	0.0257 (13)	0.0176 (11)	0.0286 (12)	−0.0038 (9)	−0.0047 (9)	0.0022 (9)
C10	0.0244 (12)	0.0165 (11)	0.0255 (12)	−0.0059 (9)	−0.0039 (9)	0.0014 (9)
C11	0.0223 (12)	0.0251 (12)	0.0257 (12)	−0.0112 (9)	−0.0035 (9)	0.0010 (9)
C12	0.0247 (13)	0.0309 (13)	0.0260 (12)	−0.0071 (10)	−0.0115 (9)	0.0014 (10)
C13	0.0311 (14)	0.0314 (13)	0.0214 (11)	−0.0115 (11)	−0.0042 (9)	−0.0010 (9)
C14	0.0177 (11)	0.0234 (12)	0.0281 (12)	−0.0048 (9)	−0.0029 (9)	−0.0034 (9)
C15	0.0526 (18)	0.0329 (14)	0.0337 (14)	−0.0073 (12)	−0.0183 (12)	−0.0059 (11)
C16	0.0327 (15)	0.0327 (14)	0.0396 (14)	−0.0087 (11)	0.0029 (11)	−0.0133 (11)
C17	0.0388 (16)	0.0217 (12)	0.0444 (15)	−0.0004 (11)	−0.0100 (12)	−0.0042 (11)
C18	0.0478 (18)	0.0217 (12)	0.0399 (15)	−0.0153 (12)	0.0001 (12)	−0.0013 (11)
C19	0.0288 (15)	0.0307 (14)	0.0603 (18)	−0.0083 (11)	−0.0126 (12)	−0.0149 (13)

### Geometric parameters (Å, °)

**Table:**

Fe1—C10	2.032 (2)	C7—C8	1.330 (3)
Fe1—C14	2.043 (2)	C7—H7	0.9300
Fe1—C15	2.045 (3)	C8—C9	1.476 (3)
Fe1—C11	2.049 (2)	C8—H8	0.9300
Fe1—C19	2.052 (3)	C9—C10	1.477 (3)
Fe1—C17	2.052 (2)	C10—C14	1.430 (3)
Fe1—C16	2.053 (3)	C10—C11	1.443 (3)
Fe1—C18	2.058 (2)	C11—C12	1.428 (3)
Fe1—C12	2.063 (2)	C11—H11	0.9300
Fe1—C13	2.063 (2)	C12—C13	1.426 (4)
O1—N1	1.228 (3)	C12—H12	0.9300
O2—N1	1.222 (3)	C13—C14	1.417 (3)
O3—C9	1.226 (3)	C13—H13	0.9300
N1—C4	1.473 (3)	C14—H14	0.9300
C1—C2	1.380 (4)	C15—C16	1.421 (4)
C1—C6	1.399 (3)	C15—C19	1.422 (4)
C1—H1A	0.9300	C15—H15	0.9300
C2—C3	1.380 (4)	C16—C17	1.418 (4)
C2—H2	0.9300	C16—H16	0.9300
C3—C4	1.375 (4)	C17—C18	1.406 (4)
C3—H3	0.9300	C17—H17	0.9300
C4—C5	1.394 (3)	C18—C19	1.428 (4)
C5—C6	1.398 (3)	C18—H18	0.9300
C5—H5	0.9300	C19—H19	0.9300
C6—C7	1.472 (3)		
			
C10—Fe1—C14	41.07 (9)	C8—C7—H7	117.1
C10—Fe1—C15	107.72 (10)	C6—C7—H7	117.1
C14—Fe1—C15	122.37 (11)	C7—C8—C9	122.6 (2)
C10—Fe1—C11	41.42 (9)	C7—C8—H8	118.7
C14—Fe1—C11	69.09 (9)	C9—C8—H8	118.7
C15—Fe1—C11	124.25 (11)	O3—C9—C8	121.9 (2)
C10—Fe1—C19	123.33 (11)	O3—C9—C10	121.0 (2)
C14—Fe1—C19	107.08 (10)	C8—C9—C10	117.0 (2)
C15—Fe1—C19	40.62 (12)	C14—C10—C11	107.73 (19)
C11—Fe1—C19	160.64 (12)	C14—C10—C9	124.5 (2)
C11—Fe1—C19	160.64 (12)	C11—C10—C9	127.6 (2)
C10—Fe1—C17	158.56 (11)	C14—C10—Fe1	69.87 (12)
C14—Fe1—C17	159.17 (11)	C11—C10—Fe1	69.92 (12)
C15—Fe1—C17	67.99 (11)	C9—C10—Fe1	121.58 (16)
C11—Fe1—C17	122.28 (11)	C12—C11—C10	107.3 (2)
C19—Fe1—C17	68.03 (11)	C12—C11—Fe1	70.20 (13)
C10—Fe1—C16	122.67 (10)	C10—C11—Fe1	68.66 (13)
C14—Fe1—C16	158.58 (11)	C12—C11—H11	126.4
C15—Fe1—C16	40.57 (12)	C10—C11—H11	126.4
C11—Fe1—C16	107.96 (10)	Fe1—C11—H11	126.3
C19—Fe1—C16	68.28 (11)	C13—C12—C11	108.4 (2)
C17—Fe1—C16	40.42 (11)	C13—C12—Fe1	69.79 (13)
C10—Fe1—C18	159.88 (12)	C11—C12—Fe1	69.17 (13)
C14—Fe1—C18	123.21 (10)	C13—C12—H12	125.8
C15—Fe1—C18	68.05 (11)	C11—C12—H12	125.8
C11—Fe1—C18	157.27 (11)	Fe1—C12—H12	126.8
C19—Fe1—C18	40.67 (11)	C14—C13—C12	108.3 (2)
C17—Fe1—C18	40.00 (12)	C14—C13—Fe1	69.06 (13)
C16—Fe1—C18	67.80 (11)	C12—C13—Fe1	69.78 (14)
C10—Fe1—C12	68.75 (9)	C14—C13—H13	125.9
C14—Fe1—C12	68.24 (9)	C12—C13—H13	125.9
C15—Fe1—C12	160.72 (12)	Fe1—C13—H13	126.9
C11—Fe1—C12	40.63 (9)	C13—C14—C10	108.3 (2)
C19—Fe1—C12	157.21 (12)	C13—C14—Fe1	70.58 (14)
C17—Fe1—C12	107.96 (11)	C10—C14—Fe1	69.06 (13)
C16—Fe1—C12	124.19 (11)	C13—C14—H14	125.8
C18—Fe1—C12	121.83 (11)	C10—C14—H14	125.8
C10—Fe1—C13	68.58 (9)	Fe1—C14—H14	126.1
C14—Fe1—C13	40.36 (9)	C16—C15—C19	108.3 (2)
C15—Fe1—C13	157.63 (12)	C16—C15—Fe1	69.99 (15)
C11—Fe1—C13	68.50 (9)	C19—C15—Fe1	69.93 (15)
C19—Fe1—C13	121.64 (11)	C16—C15—H15	125.9
C17—Fe1—C13	123.54 (10)	C19—C15—H15	125.9
C16—Fe1—C13	160.01 (12)	Fe1—C15—H15	125.8
C18—Fe1—C13	107.41 (10)	C17—C16—C15	107.6 (2)
C12—Fe1—C13	40.43 (10)	C17—C16—Fe1	69.78 (14)
O2—N1—O1	123.4 (2)	C15—C16—Fe1	69.44 (15)
O2—N1—C4	118.67 (19)	C17—C16—H16	126.2
O1—N1—C4	117.9 (2)	C15—C16—H16	126.2
C2—C1—C6	120.6 (2)	Fe1—C16—H16	126.2
C2—C1—H1A	119.7	C18—C17—C16	108.6 (2)
C6—C1—H1A	119.7	C18—C17—Fe1	70.23 (15)
C1—C2—C3	121.2 (2)	C16—C17—Fe1	69.80 (14)
C1—C2—H2	119.4	C18—C17—H17	125.7
C3—C2—H2	119.4	C16—C17—H17	125.7
C4—C3—C2	117.9 (2)	Fe1—C17—H17	125.8
C4—C3—H3	121.0	C17—C18—C19	108.2 (2)
C2—C3—H3	121.0	C17—C18—Fe1	69.77 (15)
C3—C4—C5	122.9 (2)	C19—C18—Fe1	69.42 (14)
C3—C4—N1	119.0 (2)	C17—C18—H18	125.9
C5—C4—N1	118.1 (2)	C19—C18—H18	125.9
C4—C5—C6	118.4 (2)	Fe1—C18—H18	126.5
C4—C5—H5	120.8	C15—C19—C18	107.3 (2)
C6—C5—H5	120.8	C15—C19—Fe1	69.45 (15)
C5—C6—C1	119.0 (2)	C18—C19—Fe1	69.91 (15)
C5—C6—C7	118.3 (2)	C15—C19—H19	126.3
C1—C6—C7	122.7 (2)	C18—C19—H19	126.3
C8—C7—C6	125.9 (2)	Fe1—C19—H19	125.9
			
C6—C1—C2—C3	−1.1 (4)	Fe1—C10—C14—C13	−59.89 (16)
C1—C2—C3—C4	1.0 (4)	C11—C10—C14—Fe1	59.92 (15)
C2—C3—C4—C5	−1.3 (4)	C9—C10—C14—Fe1	−115.1 (2)
C2—C3—C4—N1	177.9 (2)	C10—Fe1—C14—C13	119.44 (19)
O2—N1—C4—C3	−175.3 (2)	C15—Fe1—C14—C13	−160.87 (15)
O1—N1—C4—C3	4.8 (3)	C11—Fe1—C14—C13	81.01 (15)
O2—N1—C4—C5	3.9 (3)	C19—Fe1—C14—C13	−119.07 (15)
O1—N1—C4—C5	−176.0 (2)	C17—Fe1—C14—C13	−46.1 (3)
C3—C4—C5—C6	1.6 (4)	C16—Fe1—C14—C13	167.0 (2)
N1—C4—C5—C6	−177.6 (2)	C18—Fe1—C14—C13	−77.42 (17)
C4—C5—C6—C1	−1.6 (3)	C12—Fe1—C14—C13	37.25 (14)
C4—C5—C6—C7	176.6 (2)	C15—Fe1—C14—C10	79.69 (17)
C2—C1—C6—C5	1.4 (3)	C11—Fe1—C14—C10	−38.43 (13)
C2—C1—C6—C7	−176.7 (2)	C19—Fe1—C14—C10	121.49 (15)
C5—C6—C7—C8	−170.6 (2)	C17—Fe1—C14—C10	−165.6 (3)
C1—C6—C7—C8	7.4 (4)	C16—Fe1—C14—C10	47.6 (3)
C6—C7—C8—C9	−179.5 (2)	C18—Fe1—C14—C10	163.14 (15)
C7—C8—C9—O3	−17.4 (4)	C12—Fe1—C14—C10	−82.19 (15)
C7—C8—C9—C10	162.4 (2)	C13—Fe1—C14—C10	−119.44 (19)
O3—C9—C10—C14	−3.6 (4)	C10—Fe1—C15—C16	−119.89 (16)
C8—C9—C10—C14	176.6 (2)	C14—Fe1—C15—C16	−162.62 (15)
O3—C9—C10—C11	−177.7 (2)	C11—Fe1—C15—C16	−77.19 (18)
C8—C9—C10—C11	2.5 (3)	C19—Fe1—C15—C16	119.2 (2)
O3—C9—C10—Fe1	−89.7 (3)	C17—Fe1—C15—C16	37.75 (16)
C8—C9—C10—Fe1	90.5 (2)	C18—Fe1—C15—C16	81.04 (17)
C15—Fe1—C10—C14	−119.28 (16)	C12—Fe1—C15—C16	−43.7 (3)
C11—Fe1—C10—C14	118.65 (19)	C13—Fe1—C15—C16	163.5 (2)
C19—Fe1—C10—C14	−77.32 (17)	C10—Fe1—C15—C19	120.91 (16)
C17—Fe1—C10—C14	166.0 (2)	C14—Fe1—C15—C19	78.17 (18)
C16—Fe1—C10—C14	−161.33 (14)	C11—Fe1—C15—C19	163.61 (16)
C18—Fe1—C10—C14	−44.9 (3)	C17—Fe1—C15—C19	−81.45 (17)
C12—Fe1—C10—C14	80.84 (15)	C16—Fe1—C15—C19	−119.2 (2)
C13—Fe1—C10—C14	37.29 (13)	C18—Fe1—C15—C19	−38.16 (16)
C14—Fe1—C10—C11	−118.65 (19)	C12—Fe1—C15—C19	−162.9 (3)
C15—Fe1—C10—C11	122.08 (15)	C13—Fe1—C15—C19	44.3 (3)
C19—Fe1—C10—C11	164.03 (15)	C19—C15—C16—C17	0.1 (3)
C17—Fe1—C10—C11	47.3 (3)	Fe1—C15—C16—C17	−59.56 (18)
C16—Fe1—C10—C11	80.02 (17)	C19—C15—C16—Fe1	59.69 (18)
C18—Fe1—C10—C11	−163.5 (3)	C10—Fe1—C16—C17	−162.26 (15)
C12—Fe1—C10—C11	−37.81 (13)	C14—Fe1—C16—C17	162.6 (2)
C13—Fe1—C10—C11	−81.36 (14)	C15—Fe1—C16—C17	118.9 (2)
C14—Fe1—C10—C9	118.8 (2)	C11—Fe1—C16—C17	−119.03 (16)
C15—Fe1—C10—C9	−0.4 (2)	C19—Fe1—C16—C17	81.17 (18)
C11—Fe1—C10—C9	−122.5 (2)	C18—Fe1—C16—C17	37.17 (17)
C19—Fe1—C10—C9	41.5 (2)	C12—Fe1—C16—C17	−77.12 (18)
C17—Fe1—C10—C9	−75.2 (3)	C13—Fe1—C16—C17	−42.7 (3)
C16—Fe1—C10—C9	−42.5 (2)	C10—Fe1—C16—C15	78.85 (18)
C18—Fe1—C10—C9	74.0 (3)	C14—Fe1—C16—C15	43.7 (3)
C12—Fe1—C10—C9	−160.3 (2)	C11—Fe1—C16—C15	122.08 (16)
C13—Fe1—C10—C9	156.1 (2)	C19—Fe1—C16—C15	−37.71 (17)
C14—C10—C11—C12	−0.1 (3)	C17—Fe1—C16—C15	−118.9 (2)
C9—C10—C11—C12	174.8 (2)	C18—Fe1—C16—C15	−81.72 (18)
Fe1—C10—C11—C12	59.84 (16)	C12—Fe1—C16—C15	163.99 (15)
C14—C10—C11—Fe1	−59.89 (15)	C13—Fe1—C16—C15	−161.6 (3)
C9—C10—C11—Fe1	115.0 (2)	C15—C16—C17—C18	−0.4 (3)
C10—Fe1—C11—C12	−118.7 (2)	Fe1—C16—C17—C18	−59.75 (19)
C14—Fe1—C11—C12	−80.56 (15)	C15—C16—C17—Fe1	59.35 (18)
C15—Fe1—C11—C12	163.76 (15)	C10—Fe1—C17—C18	164.1 (2)
C19—Fe1—C11—C12	−162.6 (3)	C14—Fe1—C17—C18	−42.6 (4)
C17—Fe1—C11—C12	79.85 (17)	C15—Fe1—C17—C18	81.63 (19)
C16—Fe1—C11—C12	121.96 (16)	C11—Fe1—C17—C18	−160.80 (16)
C18—Fe1—C11—C12	46.7 (3)	C19—Fe1—C17—C18	37.66 (17)
C13—Fe1—C11—C12	−37.13 (15)	C16—Fe1—C17—C18	119.5 (2)
C14—Fe1—C11—C10	38.12 (13)	C12—Fe1—C17—C18	−118.44 (17)
C15—Fe1—C11—C10	−77.56 (17)	C13—Fe1—C17—C18	−76.6 (2)
C19—Fe1—C11—C10	−43.9 (4)	C10—Fe1—C17—C16	44.6 (3)
C17—Fe1—C11—C10	−161.47 (14)	C14—Fe1—C17—C16	−162.1 (3)
C16—Fe1—C11—C10	−119.37 (15)	C15—Fe1—C17—C16	−37.89 (17)
C18—Fe1—C11—C10	165.4 (2)	C11—Fe1—C17—C16	79.68 (18)
C12—Fe1—C11—C10	118.7 (2)	C19—Fe1—C17—C16	−81.86 (18)
C13—Fe1—C11—C10	81.55 (14)	C18—Fe1—C17—C16	−119.5 (2)
C10—C11—C12—C13	0.1 (3)	C12—Fe1—C17—C16	122.04 (16)
Fe1—C11—C12—C13	58.92 (17)	C13—Fe1—C17—C16	163.86 (15)
C10—C11—C12—Fe1	−58.86 (16)	C16—C17—C18—C19	0.5 (3)
C10—Fe1—C12—C13	−81.49 (15)	Fe1—C17—C18—C19	−58.97 (18)
C14—Fe1—C12—C13	−37.19 (14)	C16—C17—C18—Fe1	59.49 (18)
C15—Fe1—C12—C13	−164.4 (3)	C10—Fe1—C18—C17	−163.1 (2)
C11—Fe1—C12—C13	−120.0 (2)	C14—Fe1—C18—C17	163.30 (15)
C19—Fe1—C12—C13	45.1 (3)	C15—Fe1—C18—C17	−81.48 (19)
C17—Fe1—C12—C13	121.01 (15)	C11—Fe1—C18—C17	46.0 (3)
C16—Fe1—C12—C13	162.65 (15)	C19—Fe1—C18—C17	−119.6 (2)
C18—Fe1—C12—C13	79.31 (17)	C16—Fe1—C18—C17	−37.54 (16)
C10—Fe1—C12—C11	38.52 (14)	C12—Fe1—C18—C17	79.91 (19)
C14—Fe1—C12—C11	82.82 (15)	C13—Fe1—C18—C17	121.81 (17)
C15—Fe1—C12—C11	−44.4 (3)	C10—Fe1—C18—C19	−43.5 (3)
C19—Fe1—C12—C11	165.2 (2)	C14—Fe1—C18—C19	−77.11 (19)
C17—Fe1—C12—C11	−118.97 (16)	C15—Fe1—C18—C19	38.12 (17)
C16—Fe1—C12—C11	−77.34 (18)	C11—Fe1—C18—C19	165.6 (2)
C18—Fe1—C12—C11	−160.68 (15)	C17—Fe1—C18—C19	119.6 (2)
C13—Fe1—C12—C11	120.0 (2)	C16—Fe1—C18—C19	82.05 (18)
C11—C12—C13—C14	0.0 (3)	C12—Fe1—C18—C19	−160.50 (16)
Fe1—C12—C13—C14	58.49 (17)	C13—Fe1—C18—C19	−118.59 (17)
C11—C12—C13—Fe1	−58.54 (17)	C16—C15—C19—C18	0.2 (3)
C10—Fe1—C13—C14	−37.93 (13)	Fe1—C15—C19—C18	59.91 (18)
C15—Fe1—C13—C14	46.6 (3)	C16—C15—C19—Fe1	−59.73 (19)
C11—Fe1—C13—C14	−82.59 (14)	C17—C18—C19—C15	−0.4 (3)
C19—Fe1—C13—C14	78.92 (17)	Fe1—C18—C19—C15	−59.62 (18)
C17—Fe1—C13—C14	162.10 (14)	C17—C18—C19—Fe1	59.19 (19)
C16—Fe1—C13—C14	−166.1 (3)	C10—Fe1—C19—C15	−78.03 (18)
C18—Fe1—C13—C14	121.15 (15)	C14—Fe1—C19—C15	−120.14 (16)
C12—Fe1—C13—C14	−119.9 (2)	C11—Fe1—C19—C15	−44.7 (4)
C10—Fe1—C13—C12	81.97 (15)	C17—Fe1—C19—C15	81.36 (18)
C14—Fe1—C13—C12	119.9 (2)	C16—Fe1—C19—C15	37.66 (16)
C15—Fe1—C13—C12	166.5 (2)	C18—Fe1—C19—C15	118.4 (2)
C11—Fe1—C13—C12	37.30 (14)	C12—Fe1—C19—C15	165.5 (2)
C19—Fe1—C13—C12	−161.19 (15)	C13—Fe1—C19—C15	−161.82 (15)
C17—Fe1—C13—C12	−78.01 (17)	C10—Fe1—C19—C18	163.54 (15)
C16—Fe1—C13—C12	−46.2 (3)	C14—Fe1—C19—C18	121.43 (16)
C18—Fe1—C13—C12	−118.96 (15)	C15—Fe1—C19—C18	−118.4 (2)
C12—C13—C14—C10	0.0 (3)	C11—Fe1—C19—C18	−163.2 (3)
Fe1—C13—C14—C10	58.95 (16)	C17—Fe1—C19—C18	−37.07 (17)
C12—C13—C14—Fe1	−58.94 (17)	C16—Fe1—C19—C18	−80.76 (17)
C11—C10—C14—C13	0.0 (3)	C12—Fe1—C19—C18	47.1 (3)
C9—C10—C14—C13	−175.0 (2)	C13—Fe1—C19—C18	79.76 (18)

### Hydrogen-bond geometry (Å, °)

**Table:**

*D*—H···*A*	*D*—H	H···*A*	*D*···*A*	*D*—H···*A*
C7—H7···O3^i^	0.93	2.54	3.324 (3)	143.
C14—H14···O2^i^	0.93	2.67	3.377 (3)	134.
C3—H3···O1^ii^	0.93	2.66	3.278 (3)	124.
C17—H17···O1^iii^	0.93	2.68	3.539 (3)	154.

Symmetry codes: (i) −*x*+1, −*y*+1, −*z*+2; (ii) −*x*−1, −*y*+1, −*z*+3; (iii) *x*, *y*+1, *z*−1.
